# Lifespan Gyrification Trajectories of Human Brain in Healthy Individuals and Patients with Major Psychiatric Disorders

**DOI:** 10.1038/s41598-017-00582-1

**Published:** 2017-03-30

**Authors:** Bo Cao, Benson Mwangi, Ives Cavalcante Passos, Mon-Ju Wu, Zafer Keser, Giovana B. Zunta-Soares, Dianping Xu, Khader M. Hasan, Jair C. Soares

**Affiliations:** 1 Department of Psychiatry and Behavioral Sciences, The University of Texas Health Science Center at Houston, Houston, Texas USA; 2 Graduation Program in Psychiatry and Laboratory of Molecular Psychiatry, Federal University of Rio Grande do Sul, Porto Alegre, RS Brazil; 3 Department of Neurology, The University of Texas Science Center at Houston, Houston, Texas USA; 4 Department of Diagnostic and Interventional Imaging, The University of Texas Science Center at Houston, Houston, Texas USA

## Abstract

Cortical gyrification of the brain represents the folding characteristic of the cerebral cortex. How the brain cortical gyrification changes from childhood to old age in healthy human subjects is still unclear. Additionally, studies have shown regional gyrification alterations in patients with major psychiatric disorders, such as major depressive disorder (MDD), bipolar disorder (BD), and schizophrenia (SCZ). However, whether the lifespan trajectory of gyrification over the brain is altered in patients diagnosed with major psychiatric disorders is still unknown. In this study, we investigated the trajectories of gyrification in three independent cohorts based on structural brain images of 881 subjects from age 4 to 83. We discovered that the trajectory of gyrification during normal development and aging was not linear and could be modeled with a logarithmic function. We also found that the gyrification trajectories of patients with MDD, BD and SCZ were deviated from the healthy one during adulthood, indicating altered aging in the brain of these patients.

## Introduction

**Figure 1:**
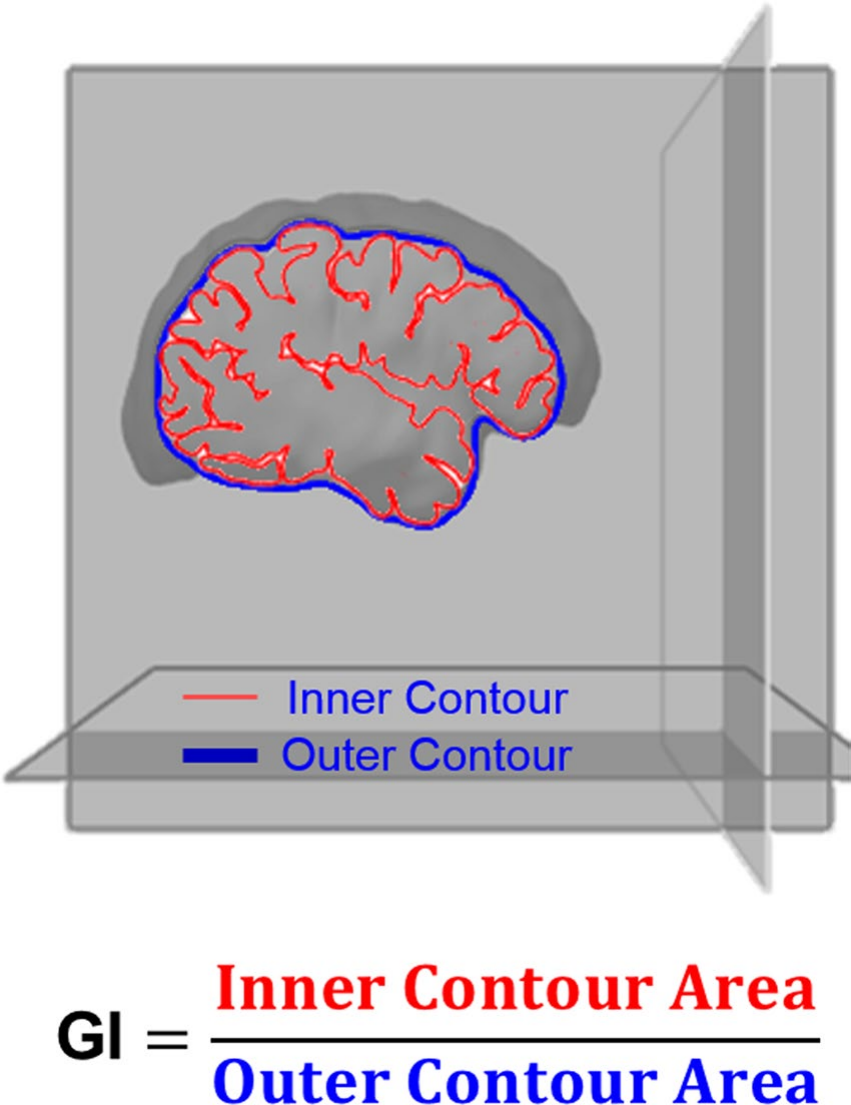
The illustration of cortical gyrification index (GI) as a ratio of the area of inner contour surface to the surface area of outer contour area.

Cortical gyrification of the brain represents the folding characteristic of the cerebral cortex, which increases the cortical surface area and thus the number of neurons in a limited cranium volume^1^. Cortical gyrification can be represented with the gyrification index (GI): a ratio of the total cortical inner surface area to the area of an outer surface that smoothly encloses the cortex^2^ (Fig. 1). The GI increases with brain mass across species^3^, and GI decreases during healthy aging in humans^4, 5^. The trajectory of brain cortical gyrification from childhood to old age in human is still unknown. Additionally, it was hypothesized that major psychiatric disorders, such as major depressive disorder (MDD), bipolar disorder (BD), and schizophrenia (SCZ), are progressive disorders associated with altered development and aging^6–13^. This hypothesis is based on the association of these disorders with higher prevalence and earlier age of onset of age-related medical conditions^14, 15^ (e.g. cardiovascular diseases and dementia), earlier cognitive decline^16^, and shortened telomere length^17^ compared to healthy controls (HC). It is possible therefore that the brain of patients with psychiatric disorders may also undergo abnormal changes during development and aging. Studies with small sample sizes have shown regional brain gyrification alterations in patients with MDD^18^, BD^19^ and SCZ^20, 21^. However, it is still unknown whether the lifespan trajectories of gyrification over the brain are altered in patients diagnosed with these psychiatric disorders and whether there are differential courses among them. In this study, we investigated the trajectory of gyrification during normal development and aging in three independent cohorts. The gyrification trajectory of healthy subjects made it possible for us to investigate the gyrification trajectories of patients with MDD, BD and SCZ with a normative refs 22–25. We hypothesized that the gyrification trajectories of patients with these major psychiatric disorders would deviate from that of the healthy subjects. Our results will fill the gap of our knowledge about the gyrification trajectory over the lifespan and provide a dynamic perspective of understanding the brain alterations in psychiatric disorders.

## Results

**Figure 2:**
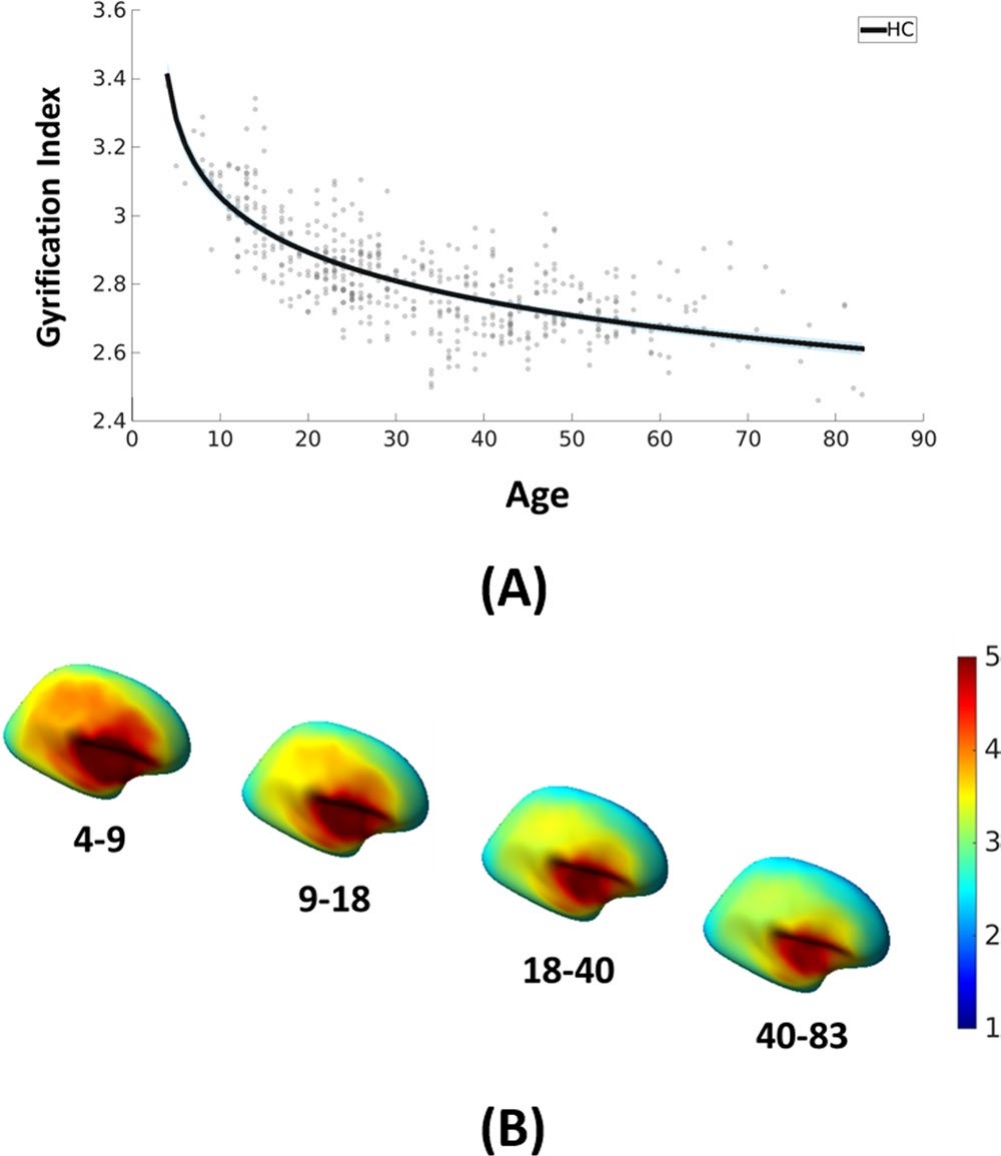
The gyrification index trajectory during normal development and aging. (**A**) The non-linear gyrification index trajectory for healthy subjects. The trajectory was well fitted with GI = *a* + *b* * *ln(age + c)*, where *a* = 3.4000, *b* = −0.1746 and c = −2.9991. Note that the thin light gray area along the black line indicates the 95% fitting confidence of the trajectory. (**B**) Brain gyrification changes over lifespan. Regional gyrification was high in childhood and adolescence, but the decrease rate was also high during these periods. Overall gyrification indices across the brain were low in adulthood, but the decrease rate was also low. The age ranges were chosen to best represent the gyrification changes. Only lateral view is shown, because the gyrification changes of medial regions over the lifespan was much less than the lateral regions (see Fig. S3). The colors represent the GI values.

The demographic information of the three independent cohorts is summarized in Table 1. In total, we have 510 HC, 95 patients with MDD, 151 patients with BD-I and 125 patients with SCZ, from age 4 to 83. The GI trajectory for HC was evaluated using the logarithmic function of age to fit the average GI trajectory (Eq. 1; see Materials and Methods), where *a* = 3.4000, *b* = −0.1746 and c = −2.9991. The coefficient of Pearson ‘s correlation between the GI estimated by Eq. 1 and the whole brain GI for the HC from all the three cohorts was r = 0.75 (p < 0.0001), accounting for 56.4% of the total variance in the average GI of all HC. Specifically, the GI estimated by Eq. 1 could account for 53.8% (r = 0.73; p < 0.0001), 66.2% (r = 0.81; p < 0.0001) and 28.3% (r = 0.53; p < 0.0001) variance of SA, NKI and COBRE samples, respectively. The difference in the variance explained by the logarithmic function could at least partly be attributed to the individual differences within HC of each cohort, e.g., COBRE HC showed higher individual difference than the other two cohorts (Supplementary Materials Fig. S2). The gyrification indices across the brain decreased over the lifespan (Fig. 2B; Supplementary Materials Fig. S3). Regional brain gyrification indices were generally higher during childhood and adolescence than adulthood, but the decrease rate was also more dramatic before adulthood.

**Table 1:** Demographic information of all subjects.

Characteristic	HC (n = 510)	MDD (n = 95)	BD-I (n = 151)	SCZ (n = 125)	F/X^2^	*P* value
Sample						
SA	225	95	151	—		
NKI	139	—	—	—		
COBRE	146	—	—	125		
Age, y*					4.83	0.0024
SA	8–65	9–62	8–68	—		
	27.7 ± 14.2	36.5 ± 16.5	32.1 ± 16.1	—		
NKI	4–83	—	—	19–64		
	33.6 ± 20.1	—	—	38.4 ± 13.0		
COBRE	18–63					
	38.5 ± 11.5					
Gender**					15.32	<0.001
Male	57.5%(293)	36.8%(35)	45.0%(68)	78.4%(98)		
Female	42.5%(217)	63.2%(60)	55.0%(83)	21.6%(27)		

*Mean and standard deviation, p value according to ANOVA; **Relative (%) and absolute (n) frequencies; p value according to χ^2^ test; Abbreviations: BD-I, Bipolar 1 Disorder; COBRE, Center of Biomedical Research Excellence; HC, Healthy Control; MDD, Major Depressive Disorder; NKI, Nathan Kline Institute; SA, San Antonio; SCZ, Schizophrenia.

**Figure 3:**
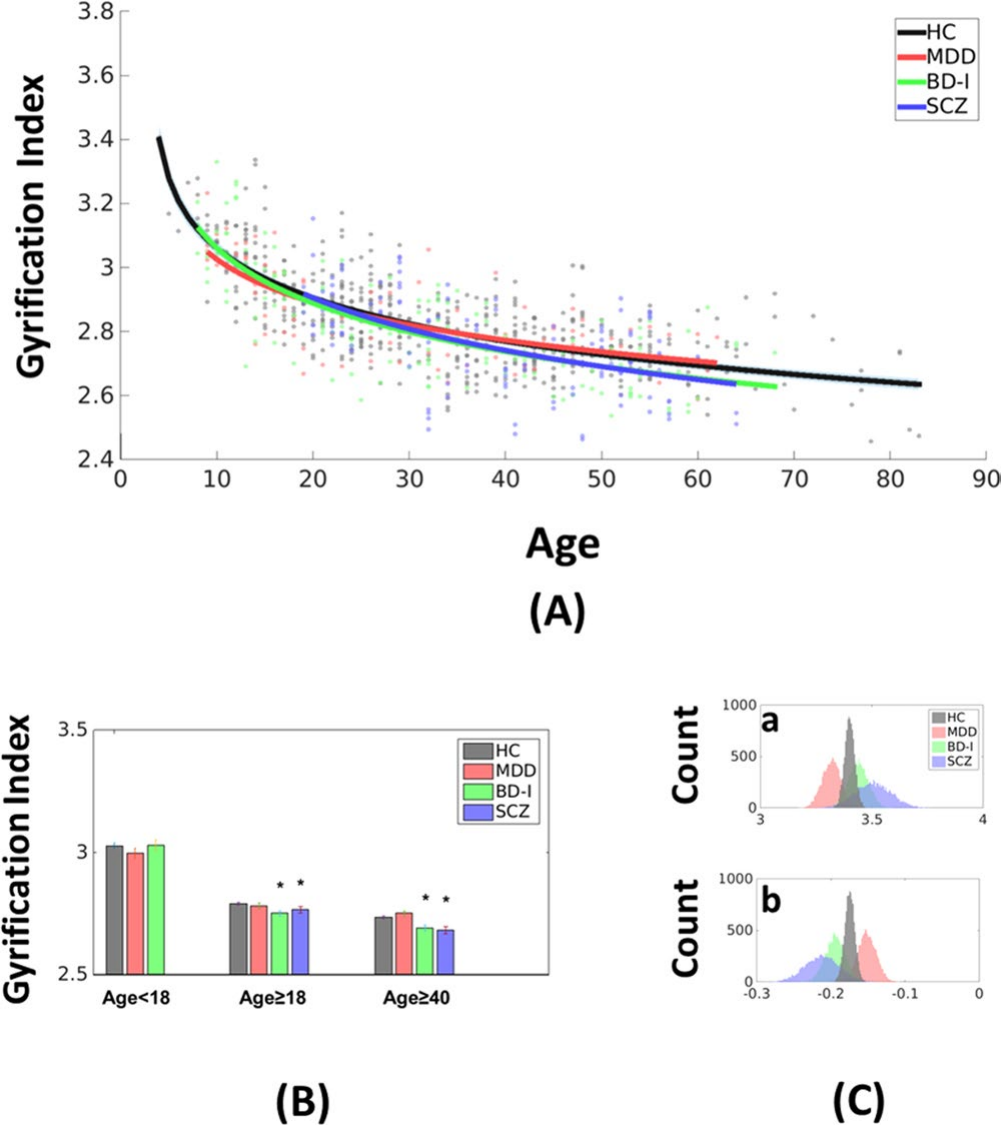
Abnormal gyrification trajectories in patients with major psychiatric disorders. (**A**) Patients with MDD, BD-I and SCZ showed deviated gyrification index (GI) trajectories from the trajectory of healthy subjects. Note that the thin light gray area along the black line indicates the 95% fitting confidence of the healthy trajectory. (**B**) The whole brain GI of healthy subjects and all the psychiatric groups in different age ranges. Patients with bipolar disorder and schizophrenia showed lower GI in the adulthood, especially after the age of 40. (**C**) Resampling results confirmed that the parameters of the fitting function for the GI trajectories of patients with psychiatric disorders were different from that of healthy subjects. Abbreviations: MDD, Major Depressive Disorder; BD-I, Bipolar 1 Disorder; SCZ, Schizophrenia; HC, healthy controls.

The GI trajectories of patients with MDD, BD-I and SCZ were estimated individually with Eq. 1 (Fig. 3A). The GI estimated by Eq. 1 accounted for 54.8% (r = 0.74; p < 0.0001), 60.9% (r = 0.78; p < 0.0001) and 33.8% (r = 0.58; p < 0.0001) variance of MDD, BD-I and SCZ patients, respectively. BD-I (p = 0.0009) and SCZ (p = 0.0427) patients showed marked decrease of GI during adulthood, especially after the age of 40 (BD-I, p = 0.0021; SCZ, p = 0.0004), compared to HC (Fig. 3B).

To confirm the GI trajectories of patients with psychiatric disorders are different from the GI trajectory of healthy subjects, we estimated the parameters of Eq. 1 for each diagnosis group using resampling technique within each group. The fitting parameters, *a* and *b*, were not different across the HC in the three cohorts (Fig. S4). We found that the parameter *a* was lower than HC in MDD (Cohen’s d = −1.68; p < 0.0001) and higher in BD-I (Cohen’s d = 0.81; p < 0.0001) and SCZ (Cohen’s d = 1.22; p < 0.0001), while the parameter *b*, which indicated the decrease rate of GI, was higher (less negative) than HC in MDD (Cohen’s d = 1.66; p < 0.0001) and lower (more negative) in BD-I (Cohen’s d = −1.35; p < 0.0001) and SCZ (Cohen’s d = −1.48; p < 0.0001) than HC (Fig. 3C). These results showed that the GI of BD-I and SCZ decreased faster than HC during aging.

The gyrification indices in multiple brain regions were significantly lower in the patients with BD-I and SCZ than HC during aging after the age of 40 (Fig. S6). No significant difference was found in MDD patients. The group-level comparison was performed at each vertex of the cortical surface. Only vertices with p values lower than the threshold p values obtained at a false discovery rate (FDR)^26, 27^ of 0.05 were shown. No further clustering adjustment was performed. To confirm the validity and uniformity of the combined HC sample, the HC of each of the three cohorts was compared to the combined HC sample, and no vertex was significantly different (Fig. S5). We also compared each of the patient samples to the corresponding HC sample within each cohort instead of the combined HC sample, and the results were similar to but less sensitive than the results with all HC (see Supplementary Materials Fig. S7).

## Discussion

To our best knowledge, this is the first *in vivo* study to show the cortical gyrification index trajectory of the human brain over the lifespan, from age 4 to 83. During this age range, the GI trajectory follows a logarithmic function of age. This is an important advancement of our knowledge on human brain development and aging, as previous studies showed a linear decrease of GI during the adulthood^4, 28^. Based on *post-mortem* studies, the gyrification increases after birth^29, 30^, which was confirmed by a recent longitudinal imaging study on children before the age of two^31^. However, when the gyrification of human brain reaches its peak during development is still unknown. Although there have been studies suggesting that the brain complexity keeps increasing until teenage^32^ and certain brain regions such as entorhinal cortex may have increased thickness until the age of 30^33^, our study suggests that the GI starts to decrease already around the age of four. The cellular mechanisms underlying the gyrification are still unclear^34^, although theories have been raised based on mechanical tension^35^, stress-dependent folding and differential growth^36, 37^, regulated radial and tangential expansion^38^, axonal pushing^39^ and minimization of effective free energy associated with cortical shape^3, 30^. Most of these theories focused on the brain development during the young age, and the trajectory observed in the present study may be associated with different mechanisms. Thus, the gyrification trajectory established in this study will be valuable information that needs to be considered in the future gyrification theories considering both brain development and aging.

Our findings revealed abnormal gyrification trajectories in patients with major psychiatric disorders. The GI in patients with BD-I and SCZ decreased faster than HC during aging. The brain regions with altered gyrification indices in BD-I and SCZ are consistent with several previous brain imaging studies investigating them individually^19, 20, 40, 41^. Patients with SCZ showed a significant decrease of GI in the dorsolateral prefrontal cortex, anterior cingulate cortex and supra-marginal cortex. The prefrontal cortex is known to be crucial for executive function^42^ and working memory^43^. These important cognitive functions are impaired in SCZ^44^, which has been linked to the dysfunction of dorsolateral prefrontal cortex, such as low activity, low N-acetylaspartate concentration and abnormal dopamine metabolisms^45–47^. The anterior cingulate cortex is the key region for conflict monitoring, motivation modulation and mood regulation^48–50^, the pathology of which has been shown in mood disorders and schizophrenia^51–54^. The abnormally decreased gyrification indices of these regions are in line with the altered cognitive monitoring and executive control^55^ in SCZ. The GI decrease in patients with BD-I was less extensive compared to patients with SCZ. However, lower GI in BD-I than HC in inferior frontal regions was consistent with previous studies on cortical gray matters^56^. We also found that the gyrification trajectory of patients with MDD might be different from HC, as well as from BD-I and SCZ, but no brain region showed significant alteration of gyrification in MDD. In fact, several studies with small sample size showed higher gyrification in MDD than in healthy controls^57, 58^, while some showed lower^18, 59^, which could be due to differences in genes or brain connections^57, 59^. Further studies will be necessary to clarify these inconsistent results. However, our results did show a possible decrease of gyrification index in MDD at a young age, which can be confirmed with future longitudinal studies focusing on pediatric MDD. The more significant decrease of gyrification in BD and SCZ compared to MDD could also be related to the possible damages due to manic episodes in BD and positive symptoms in SCZ indicated in the neuroprogression theory of BD and SCZ^60, 61^. In addition, this is in line with recent studies, which suggested that the number of manic episodes seems to be the clinical marker more robustly associated with brain changes and neuroprogression in BD^61^. Given that the psychiatric disorders have been a major burden^62^ in this aging world^63^, brain markers of abnormal aging, such as the gyrification index, may help us understand the mechanisms of brain aging, evaluate future strategies to slow down the degenerative process and relieve the burden caused by these major psychiatric disorders.

Some limitations should be considered in our study. The GI trajectory over the lifespan was developed using only cross-sectional data, and longitudinal data with multiple follow-ups were necessary to map the individual GI trajectory and to compare the individual differences of the GI trajectory during aging. The GI obtained with the automated algorithm was slightly higher than the ones in the previous studies^31, 64^. However, a recent study showed a similar range of GI using the same automated algorithm^65^. Our study covered a large age range of human lifespan from 4 to 83, but the GI trajectory before the age of four was not included. This was due to multiple limitations, e.g., the data that were publicly available over the lifespan usually did not include children below the age of four; there were challenges to perform MRI scans on young children due to the head motion; and validation of the current automated algorithm on young children was still necessary.

In summary, the present study demonstrated for the first time that the gyrification of the human brain *in vivo* decreased non-linearly from the age of 4 to 83 and this process could be modeled with a logarithmic function of age. The results were consistent across three independent cohorts. Moreover, by comparing the gyrification trajectories of healthy subjects and patients diagnosed with major psychiatric disorders, such as major depressive disorder, bipolar disorder, and schizophrenia, we provided consistent evidence that these disorders were associated with abnormal gyrification during aging. These results will advance our knowledge about how our brain changes during normal and abnormal aging.

## Materials and Methods

### Samples

Structural brain images from 881 subjects were collected with T1-weighted magnetic resonance imaging (MRI) from three independent cohorts: a sample collected at San Antonio (the SA sample), a sample collected at Nathan Kline Institute - Rockland (the NKI sample)^66^ and a sample collected by the Centers of Biomedical Research Excellence (the COBRE sample). The latter two samples were publicly available. All patients completed the Structured Clinical Interview for DSM-IV Axis I Disorders (SCID) and met the corresponding DSM-IV criteria for MDD, bipolar 1 disorder (BD-I) or SCZ. Healthy controls (HC) had no current condition or history of psychiatric dysfunctions. All subjects had no history of neurological diseases. Signed informed consent have been obtained from all the subjects. No identifiable image from a specific subject was used for publication. The study protocols were approved by the Institutional Review Boards at the University of Texas at San Antonio in accordance with their guidelines and regulations.

### Image Acquisition and Processing

The SA sample was all acquired at one Philips 1.5 Tesla MRI scanner (Philips Medical System, Andover, MA, USA) with a three-dimensional axial fast field echo sequence with the following parameters: repetition time (TR) = 24 ms, echo time (TE) = 5 ms, flip angle = 40°, field of view (FOV) = 256 mm, slice thickness = 1 mm, matrix size = 256 × 256 and 150 slices. All scans were visually inspected to rule out gross artifacts. The NKI sample was acquired at one 3 Tesla Siemens Magnetom TrioTim syngo scanner with a three-dimensional magnetization-prepared radio-frequency pulses and rapid gradient-echo (MPRAGE) sequence with the following parameters: TR = 2500 ms, TE = 3.5 ms, flip angle = 8°, FOV = 256 mm, slice thickness = 1 mm, matrix size = 256 × 256 and 192 slices (see http://fcon_1000.projects.nitrc.org/indi/pro/nki.html). The COBRE sample was acquired at one 3 Tesla Siemens Magnetom TrioTim syngo scanner with a three-dimensional multi-echo (ME-MPRAGE) sequence with the following parameters: TR = 2530 ms, TE = 1.64, 3.5, 5.36, 7.22, 9.08 ms, flip angle = 7°, FOV = 256 mm, slice thickness = 1 mm, matrix size = 256 × 256 and 176 slices (see http://fcon_1000.projects.nitrc.org/indi/retro/cobre.html).

The structural brain images were preprocessed and the cortical surface of each brain was reconstructed with Freesurfer^67, 68^ (version 5.3, http://surfer.nmr.mgh.harvard.edu/). The reconstructed images were visually inspected by the authors to exclude the apparent reconstruction errors. The local gyrification index (GI) at each vertex of the reconstructed cortical surface mesh was calculated using the toolbox in Freesurfer with default settings^69^. Briefly, a circular region of interest was delineated on the outer surface, and its corresponding region of interest on the inner cortical surface was identified using a matching algorithm as described elsewhere^70^ and the ratio between the folded inner cortical surface and its corresponding exposed outer surface was calculated as GI. The resulted GI values of each subject were smoothly sampled (Gaussian kernel, 10 mm) onto an average template provided by Freesurfer (fsaverage; 163842 vertices), so that cross individual comparison could be performed. The averaged GI for each subject was also calculated to represent the gyrification of whole brain.

The effect of brain volume on GI was taken into account for by adjusting the GI values with a linear regression of intracranial volume (ICV) on GI at each vertex and the whole brain GI, because previous studies found that the GI could be correlated with the brain volume^31, 71^.

### Modeling Gyrification Trajectory as a function of Age

We compared the fitting of the whole brain GI with age from ten common mathematical functions and chose the best function based on the averaged mean squared errors (MSE). The MSE of each fitting function was calculated with the non-linear fitting functions of the Statistics and Machine Learning Toolbox in MATLAB (The MathWorks, Inc., Natick, Massachusetts, United States) using default settings. Because the COBRE sample only included adults, it was not involved in the function selection process. For SA and NKI samples, the MSE of each function was calculated based on the residuals derived from two types of validation procedures: (a) a leave-one-out cross-validation within each of the two samples and (b) a cross-sample validation, in which a fitting function was optimized for one sample and tested on the other. Thus, we had four MSEs for each function and the averaged of these MSEs was used as the fitting performance for the corresponding function as shown in Table S1. The best function was the logarithmic function with three parameters.

As a result, we used the following logarithmic function of age to fit the average GI trajectory:

1 $${\rm{GI}}=a+b\ast ln(age+c)$$

where *a* was an indicator of GI levels independent of age, *b* controlled the decrease rate of GI over age and *c* was the translational term of age. The initial value of *a* was set to 4, because the mean of the brain average GI values of all the HC subjects was around 3 and the maximum whole brain GI was near 4. The initial value of *b* was set to −1, because GI apparently decreased with age. The initial value of c was set to 0. We found that *c* was sensitive to the age range of the sample and we had varied age range in COBRE HC, MDD, BD and SCZ samples. Furthermore, *c* did not provide any information of the GI levels and trajectory shapes, and its value was consistent in SA and NKI HC samples. In order to reliably compare the GI trajectories of all the samples without changing the shape and levels of GI trajectory, we fixed the c value to −2.9991 according to the cross-validated fitting for the combined SA and NKI HC sample. Then each of the MDD, BD and SCZ groups was fitted separately.

GI was adjusted for study site by an amount of the estimated GI difference between the HC in each cohort and the combined SA and NKI HC sample at a given age [*a*_*HC*_ + *b*_*HC*_ $\ast$ *ln*(*age* + *c*)] − [*a*_*X*_ + *b*_*X*_ $\ast$ *ln*(*age* + *c*)], where X represents the sample of HC in SA, NKI and COBRE cohorts and the mean adjustment values were as small as −0.0023, 0.0043 and 0.0523, respectively. The adjustment for the COBRE cohort was larger than the other two cohorts, because the GI in the COBRE HC sample was generally lower than the HC in the other two cohorts, which could also be observed in the mean GI of adult HC in the COBRE cohort (2.748) compared to the GI of adult HC in SA (2.7938) and NKI cohorts (2.7777). To further confirm whether the adjustment introduced significant effect in the SCZ sample in the COBRE cohort, we also compared the GI over the brain of HC and SCZ within the COBRE cohorts.

Although the samples showed different distributions of males and females (see also^72^), which might have an effect on the GI trajectory of the samples with major psychiatric disorders, we found that effect of gender on GI was negligible after GI was adjusted by the ICV (Supplementary Materials Fig. S1). This might indicate that the gender effect on GI could be mostly explained by the brain volume. Thus, no further adjustment for gender was performed.

#### Estimating the Parameters with Resampling

In order to quantify the difference between the GI trajectories of HC, MDD, BD-I and SCZ, it was necessary to estimate the distributions of fitting parameters. We utilized the resampling technique. Briefly, for each of the HC, MDD, BD and SCZ samples, the sample was re-sampled 10000 times and the fitting was performed for each of the 10000 samples. In order to stratify the age distribution during the resampling, each sample was divided into four age blocks: 4–9, 9–18, 18–40 and 40–83. During each iteration of the resampling, 50% of the subjects in each age block were selected without duplicate. Thus, we had 10000 sets of parameters for each sample, and it was then possible to estimate the distributions of the fitting parameters in different samples.

## Electronic supplementary material

Supplementary Materials
