# Supplementary material for: Lifespan Gyrification Trajectories of Human Brain in Healthy Individuals and Patients with Major Psychiatric Disorders
Source: Sci Rep. 2017 Mar 30;7:511. doi: 10.1038/s41598-017-00582-1 (PMC5428697; doi:10.1038/s41598-017-00582-1)
Supplement: Supplementary file 1 — Supplementary Materials [file 41598_2017_582_MOESM1_ESM.pdf]

Lifespan Gyrification Trajectories of Human Brain in Healthy Individuals and Patients with Major Psychiatric Disorders

Supplementary Materials

Bo Cao[1], Benson Mwangi[1], Ives Cavalcante Passos[2], Mon-Ju Wu[1], Zafer Keser[3], Giovana B. Zunta-Soares[1], Dianping Xu, Khader M. Hasan[4], Jair C. Soares[1]

Table S1. Comparisons between different fitting functions

Mathematical Function	a_0	b_0	c_0	d_0	Average MSE
**$a+b^x$**	3	0.9	-	-	0.0102
**$a+b^{x+c}$**	3	0.9	0	-	0.0098
**a+bx**	4	-1	-	-	0.0126
**$a+bx+cx^2$**	4	0	-1	-	0.0123
**$a+bx+cx^2+dx^3$**	4	0	0	-1	0.0099
**$a+x^b$**	3	-0.1	-	-	0.0125
**$a+bx^c$**	3	1	-0.1	-	0.0099
**$a+b(x+c)^d$**	3	1	0	-0.1	0.0101
**a+b*ln(x)**	4	-1	-	-	0.0098
**a+b*ln(x+c)**	4	-1	0	-	0.0088

a_0, b_0, c_0 and d_0 are the initial values of a, b, c, and d. MSE, mean squared errors.

Lifespan gyrification trajectories in males and females

Lifespan gyrification trajectories in healthy males and females are shown in in Figure S1. Males had higher GI than females over the lifespan. However, this effect was significantly decreased to a negligible level, especially during the adulthood, after the GI was corrected with ICV. Thus, the higher GI in males could be mostly accounted by the higher ICV in males than females.

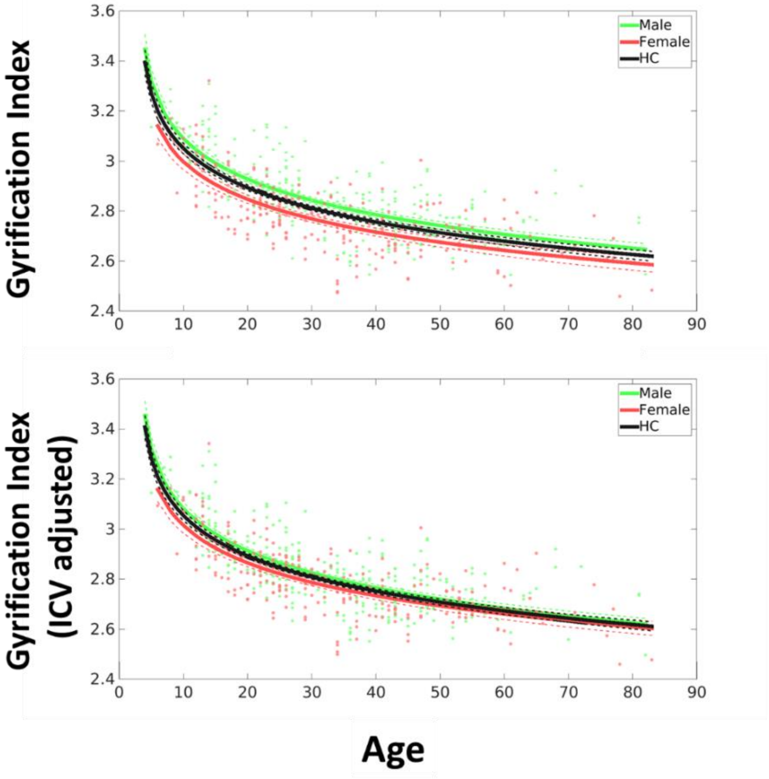

Figure S1. Gender effect on GI was negligible after GI was adjusted by ICV.

Lifespan gyrification indices across the brain

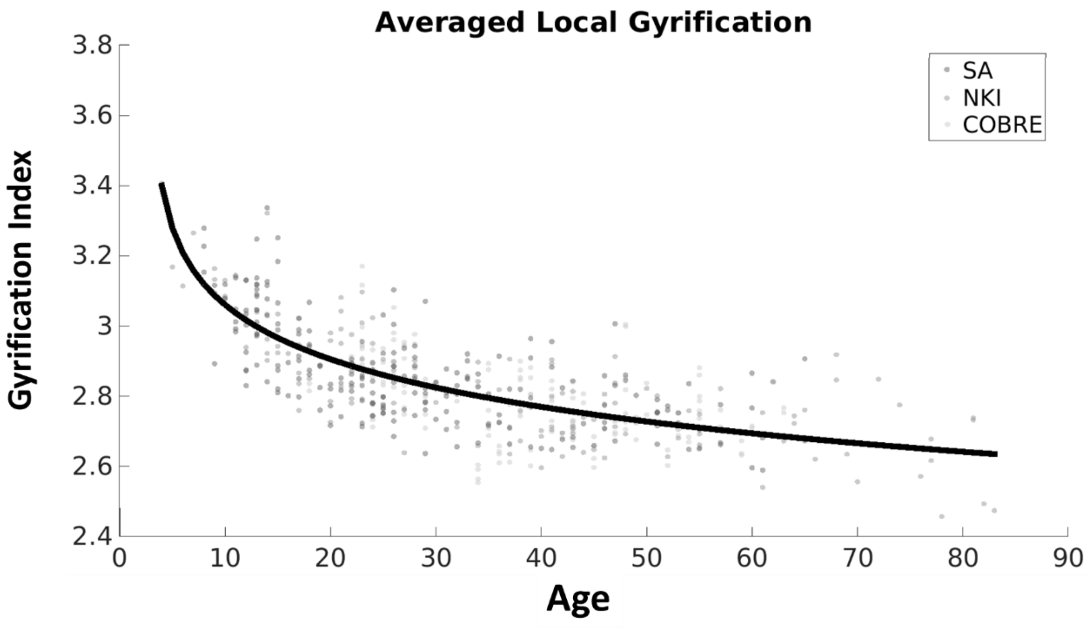

Figure S2. Lifespan gyrification indices of healthy controls (HC) from three cohorts. The COBRE HC (light grey) showed higher individual difference than the HC from the other two cohorts. The solid black line showed the logarithmic fitting for the combined HC sample.

Lifespan gyrification indices across the brain in adulthood are shown in a finer age scale

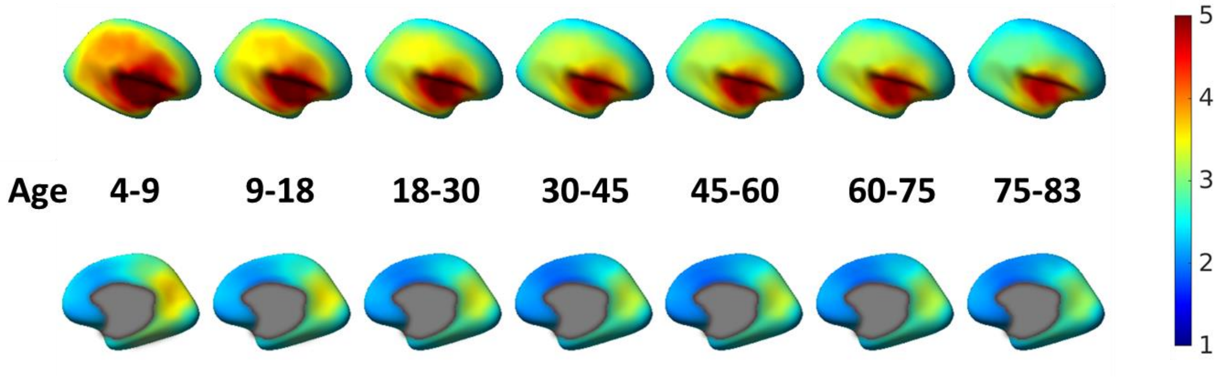

Figure S3. Lifespan gyrification indices across the brain. Brain gyrification index decrease were less compared to the changes before adulthood.

The distribution of fitting parameters for the HC in the three cohorts

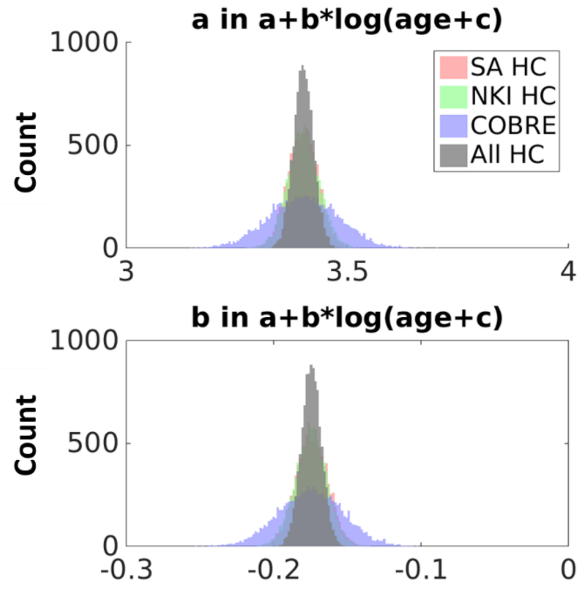

Figure S4. The distribution of fitting parameters for the HC in the three cohorts. The fitting parameters, a and b, were not different across the HC in the three cohorts

Brain gyrification in the HC of three cohorts compared to all HC

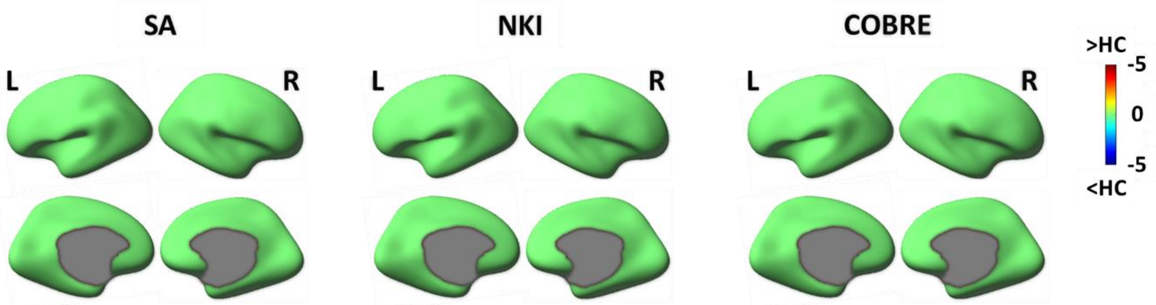

Figure S5. Brain gyrification in the HC of three cohorts compared to all HC. The group-level comparisons were the same with those of patient groups vs. all HC. The p values were thresholded with the p values controlled for a false discovery rate of 0.05. No vertex was significantly different in any of the HC samples compared to the combined HC sample.

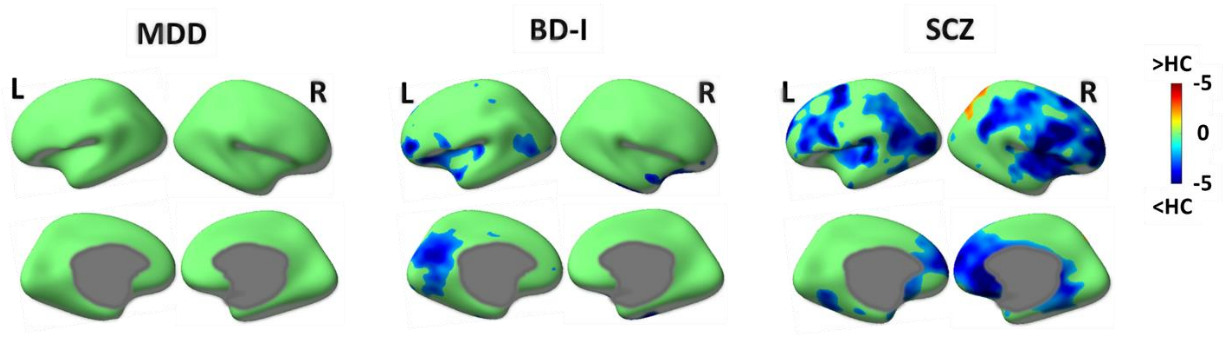

Figure S6. Abnormal gyrification changes in multiple brain regions in patient with major psychiatric disorders during aging (age 40 to 83). The color represents the logarithm of the p value for each vertex, with colors towards red indicating greater GI than healthy controls (HC) and colors towards blue indicating lower GI than HC. The central grey region in the medial views shows the non-cortical region. L and R represent left and right hemisphere, respectively. Abbreviations: MDD, Major Depressive Disorder; BD-I, Bipolar 1 Disorder; SCZ, Schizophrenia.

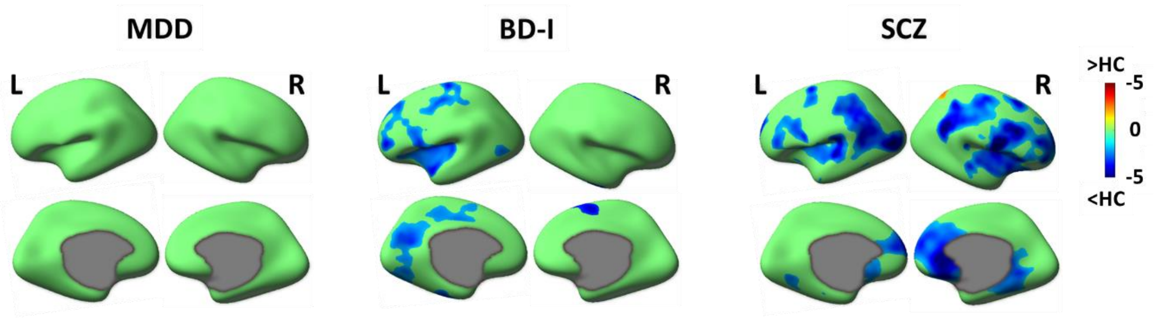

Figure S7. Brain gyrification in MDD, BD-I and SCZ compared to the HC in the same cohort. The group-level comparisons were the same with those of patient groups vs. all HC. The p values were thresholded with the p values controlled for a false discovery rate of 0.05. The results were similar compared to Figure 4 in the main text.
